# Diagnostic and Surgical Planning Value of Computed Tomography (CT) in Adult Ankle Fractures: A Narrative Review

**DOI:** 10.7759/cureus.98749

**Published:** 2025-12-08

**Authors:** Aishwarya Ghosh

**Affiliations:** 1 Trauma and Orthopaedics, St George's Hospital, London, GBR

**Keywords:** acr appropriateness criteria, ankle fracture, computed tomography, diagnostic accuracy, nice ng38, posterior malleolus, radiography, surgical planning, syndesmosis

## Abstract

Ankle fractures are common adult injuries with complex patterns often inadequately characterised by plain radiographs alone. Computed tomography (CT) has been shown to improve diagnostic accuracy and inform surgical planning, though routine use remains limited due to cautious guidelines. This narrative review synthesises evidence from 20 unique studies (2010-2025) to compare CT with X-rays in adult ankle fractures and evaluate diagnostic precision, fracture classification, and changes in operative strategy. CT consistently enhanced the detection of complex and occult fractures, particularly posterior malleolar and syndesmotic injuries missed on X-rays, with fracture classification altered in up to 44% of cases and surgical management changed in approximately 25%, especially for tri-malleolar and posterior malleolus fractures. Despite these benefits, the evidence base is predominantly level III-IV, with limited data on cost-effectiveness or long-term functional outcomes. In alignment with National Institute for Health and Care Excellence NG38 and the American College of Radiology Appropriateness Criteria (2023), the review concludes that while CT improves diagnostic and surgical decision-making for complex ankle fractures, its routine use lacks proven patient centred benefit; therefore, a selective CT algorithm is proposed to help clinicians identify fracture patterns where CT is most likely to influence management, balancing clinical value, cost, and radiation exposure.

## Introduction and background

Ankle fractures are the second most common fracture in adults (9%) after neck of femur fractures, with an incidence rate of around 180 per 100,000 people annually [1]. They are sustained by a variety of mechanisms and affect all ages, genders, and races [1]. Furthermore, nearly a third of all ankle fractures (32%) involve two or more malleoli, altering joint articulation and congruity.

Appropriate and accurate imaging is essential for timely management and serves as the foundation for successful patient outcomes. Plain radiographs or X-rays (anterior-posterior/mortise and lateral views) remain essential, first-line investigations for the early assessment of all suspected ankle fractures, but particularly after reduction, to confirm realignment [2,3]. However, complex fracture patterns, for example, posterior malleolus involvement, may be underappreciated on X-ray alone, and if missed, potentially may lead to suboptimal surgical planning or fixation, longer surgery, revision surgery, post-traumatic arthritis, and poor functional outcomes for the patient [1]. Anatomical reduction of the fracture and restoration of joint congruity are essential; even a 1 mm lateral shift of the talus reduces the contact area of the ankle by 42%, causing high contact stress and increasing the risk of joint degeneration [4].

Computed tomography (CT) imaging provides additional details on fracture morphology and joint involvement that can assist in surgical planning. However, its use carries additional radiation exposure (approximately 2-4 mSv per ankle scan) and cost, which must be balanced against potential clinical benefit [5].

Current guidelines reflect uncertainty regarding the use of CT in ankle fractures. The National Institute for Health and Care Excellence (NICE) NG38 report recommends X-ray as the initial imaging modality. However, it highlights the need for research to establish whether the clinical and cost-effective use of adjunctive CT improves surgical outcomes for unstable or displaced fractures [6]. Similar guidance is echoed in the 2020 American College of Radiology (ACR) Appropriateness Criteria for acute trauma to the ankle, in which CT use is selectively reserved for preoperative planning of suspected occult fractures or for persistent symptoms following a negative X-ray [7].

Evidence to date exploring the use of CT in ankle fracture diagnosis and management has been mostly heterogenous, level III-IV observational work, with no large-scale randomised controlled trials to accurately capture clinical and cost-effective outcomes or to guide when to use CT.

Therefore, this structured narrative review aims to address a clear gap highlighted in current guidance by synthesising relevant literature on three domains: the diagnostic accuracy of CT compared with X-ray, how CT findings alter surgical planning and decision-making (compared to X-rays), and situations in which CT could be selectively used. The goal is to provide clinicians with a practical, evidence-based framework to support imaging decisions in adult ankle fractures, balancing patient benefit, cost, and radiation exposure.

## Review

Methodology

Studies relevant to this topic were reviewed, focusing on the role of CT relative to plain radiographs (X-rays) in diagnosing, classifying, and planning surgery for adult ankle fractures. To capture contemporary evidence, we explored literature published between January 2010 and October 2025 using PubMed and consulted guidelines from relevant professional bodies where applicable. Key concepts included ankle fractures, involvement of the posterior malleolus, and imaging modalities (CT and radiography) in relation to diagnostic accuracy and surgical planning.

Core search terms included combinations of “ankle fracture” OR “posterior malleolus” OR “malleolar fracture” OR “bimalleolar” OR “trimalleolar” OR “syndesmotic injury” AND (“computed tomography” OR “CT scan”) AND (“radiography” OR “plain radiograph” OR “X-ray”) AND (surgical OR preoperative OR planning OR management OR “change in management” OR “diagnostic accuracy”).

Inclusion criteria were any studies that reported direct comparison of CT and X-ray in adult ankle or malleolar fractures and presented data on diagnostic accuracy, fracture classification, or changes in surgical planning/management. Exclusion criteria were studies involving the paediatric or poly-trauma cohorts, case reports, technical notes, or reviews lacking diagnostic, management, or outcome data, and non-human or non-English publications.

Findings from the identified literature were narratively synthesised to highlight trends in diagnostic performance and the influence of CT on surgical decision-making compared to conventional radiography.

This review is narrative in nature and does not follow a systematic review protocol. No formal risk-of-bias assessment or meta-analysis was performed. The use of a single database (PubMed) is a significant limitation, as it restricts the breadth of evidence considered. Most included studies were retrospective, level III-IV, varied in methodology, and originated from a single centre. Thus, although this structured review aims to capture the best available comparative data on CT use in ankle fracture diagnosis and surgical planning, it cannot definitively establish causal or cost-effectiveness relationships.

Results

Diagnostic Accuracy Between CT and Plain X-Rays (Detection)

Eleven studies were identified that explored the role of CT compared with plain X-rays, with varying designs and sample sizes (47 to 279 participants), and their key characteristics are summarised in Table 1 [8-18].

**Table 1:** Diagnostic accuracy studies comparing CT and plain X-rays CT: computed tomography, AOFAS: American Orthopaedic Foot and Ankle Society, OAR: Ottawa Ankle Rules, PMF: posterior malleolus fragment

Author (year)	n	Key finding (missed injuries on X-ray)	Impact on classification/planning	Level of evidence
Mansur et al. (2024) [8]	53	X-ray missed ~50% of syndesmotic lesions; CT detected 1.5× additional posterior fragments not seen on X-ray (p<0.001)	CT diagnosis changed surgical plans: increased operative indication, fixation, altered positioning, and approach (p<0.01)	III
Palmanovich et al. (2020) [9]	85	X-ray unable to distinguish posterolateral/central/medial fragment types that CT clearly delineated	CT classification suggested screw trajectory angles (21°, 7°, 28°) for surgical planning vs fixed (~20° on X-ray) for optimal fixation orientation of the posterior malleolus fragment	III
Carrozzo et al. (2018) [10]	51	Bilateral preoperative CT provided more accurate detection of syndesmotic mal-reduction compared with X-ray or single-side CT	Improved postoperative alignment and higher functional scores at 12 months (median AOFAS score 93.44 +/- 3.01) - bilateral CT improved anatomical restoration	II
Vosoughi et al. (2019) [11]	47	X-ray cannot differentiate large intra-articular (Pilon-type) vs smaller extra-articular avulsion posterior fragments	CT changed classification and thus planning - large intra-articular fractures fixed; small avulsions managed conservatively	III
Wang et al. (2013) [12]	183	Among OAR-positive but X-ray-negative cases, CT detected occult lateral malleolus fractures in 23.8% (n=5)	The OAR could reduce unnecessary X-rays by ~31.1 % by using CT for X-ray indications in cases with negative CT but clinically suspicious findings	II
Thomas et al. (2024) [13]	98	X-ray underestimated PMF size in some cases but correlated moderately with CT (r=0.724)	CT altered fixation in only 1 of 45 small PMFs (<20% on X-ray); suggests threshold-based CT use (>20% PMF width)	III
Bouche et al. (2021) [14]	60	X-ray detected 58% of posterior malleolus fragments vs 88% on CT (p<0.01)	Routine preoperative CT for bimalleolar fractures may avoid missing posterior malleolus fractures for fixation	IV
Black et al. (2021) [15]	279	X-ray unable to determine medial extension of taller posterior fragments	Suggests increased usage of preoperative CT in the setting of taller lateral height fracture (>24.5mm) on X-rays (greater likelihood of medial extension)	IV
Yeung et al. (2015) [16]	123	X-ray not reliable for syndesmosis instability; CT identified abnormal anterior tibiofibular distance predictive of instability (sensitivity 56.4 % and specificity 91.7 %) in 39 (31.7 %) cases - operatively diagnosed with syndesmosis instability	Suggests the value of CT axial measurement for predicting syndesmosis stability and fixation	IV
Szymański and Zdanowicz (2022) [17]	67	40% of pathologies (Tillaux, Pilon, loose bodies, syndesmosis) were missed on X-ray	CT is essential for complete injury mapping	IV
Robles et al. (2024). [18]	61	X-ray sensitivity 71% vs CT 100% (standard)	CT adds accuracy for identifying impaction and guiding articular surface reduction	III

Several studies reported that X-rays failed to detect a substantial proportion of injuries, with estimates ranging from approximately 23.8% to 50%, including posterior malleolar and syndesmotic injuries, intra-articular avulsions, and subtle joint impaction and articular injuries [8,9,12,14,17,18]. Robles et al. noted that both X-ray and CT are comparable for ruling out major fractures; however, the sensitivity was 29% higher in CT for joint impaction and articular injuries compared to X-ray (sensitivity of 71%) [18]. Similarly, Yeung et al. reported that CT axial measurements of an abnormal anterior tibiofibular distance had a 56.4% sensitivity and 91.7% specificity for detecting potential syndesmotic instability compared with X-rays alone [16]. Carrozzo et al. found that preoperative bilateral CT imaging of the ankles (fractured and normal) improved the accuracy of detecting syndesmotic malreduction compared with X-ray and unilateral CT alone [10]. While this cohort demonstrated improved postoperative alignment and functional outcomes (median American Orthopaedic Foot and Ankle Society (AOFAS) of 93.4 ± 3.0), these findings suggest only potential implications for longer-term function rather than direct evidence of benefit from CT itself. Collectively, these studies reinforce how CT provides detailed injury mapping, capturing patterns and fragments that may be overlooked on plain X-rays

Furthermore, CT altered fracture classification in five studies [8,9,11,13-15]. This is particularly important with posterior malleolus fractures, where fragment characteristics inform surgical management. In Mansur et al.'s study, CT identified approximately 1.5 times as many posterior malleolus fragments and associated characteristics (avulsion, displacement) as X-ray alone, resulting in increased operative indications (p=0.007) and fixation (p<0.001) [8]. Similarly, in Vosoughi et al.'s study, CT differentiated 47 postero-medial fractures into large intra-articular Pilon fragments (n=29) requiring surgical fixation vs smaller extra-articular fragments (n=18) suitable for conservative management [11]. Palmanovich et al. highlighted that while X-rays could not reliably classify posterior fragments, CT axial cuts distinguished lateral, central, and medial patterns and even suggested optimal screw trajectory angles for fixation [9]. In Bouche et al.'s study, 35 of 60 posterior malleolus fragments (58.3%) were visible on X-ray compared to 53 fragments (88.3%) on CT (p<0.01) [14]. In a multicentre retrospective study of 279 patients, Black et al. showed that CT was particularly valuable for characterising posterior fragments [15]. Fragments with a lateral height greater than 24.5-29.5 mm had a 3.1-fold increased risk of medial extension, and those with a lateral height greater than 29.5 mm had an 8.1-fold greater risk of medial extension, compared to fragments with lateral heights less than 20.5 mm (and hence likely need surgical intervention). Similarly, Thomas et al. reported a moderate correlation between X-ray and CT for posterior malleolus fragment size (r=0.724); however, CT altered fixation strategy in fragments >20% of the joint surface, demonstrating threshold-based utility [13]. Combined, these findings underscore that CT not only improves the detection of subtle or complex fracture features but also provides critical information that informs surgical planning, reinforcing its diagnostic value in ankle fracture management.

Evaluating Change in Surgical Management Plan With CT Imaging

Nine studies were identified that examined the impact of CT imaging on surgical management decisions, with sample sizes ranging from 20 to 288 patients, summarised in Table 2 [19-27].

**Table 2:** Studies evaluating change in surgical management plan with CT imaging CT: computed tomography, TCF: talar‑calcaneal fracture

Author (year)	n	Fracture type	Surgical plan change after CT (yes/no; % changed)	Key findings	Level of evidence
Kumar et al. (2018) [19]	58	>1 malleolus fracture	Yes - 13 (23.2%) cases	Fixation of the posterior malleolus, then the lateral malleolus, in 4 cases	IV
Meijer et al. (2016) [20]	31	With posterior malleolus	Yes - 7 (23%) cases	Influenced the surgical approach and fragment size	III
Donohoe et al. (2017) [21]	25	With posterior malleolus	Yes - 44% cases	CT improved subsequent fixation	III
Massri‑Pugin et al (2025) [22]	83	TCF in adults	Yes - 12.5% cases and classification (Rammelt) changed in 69.1% of cases	Patient position changed in 32.1%, fixation type in 43.8%, and surgical approach in 35.3% of cases	III
Kalantar et al. (2024) [23]	163	Foot and ankle trauma	Yes - 38 (23.3%) cases	CT revealed additional findings; X-ray missed ≥1 fracture in 39.9%	IV
Sheikh et al. (2020) [24]	20	With posterior malleolus	Yes - 32.7% of cases	Posterior malleolus fixed in 25.6%, syndesmosis fixed in 16.6%	III
Leung et al. (2016) [25]	69	Ankle fractures in adults	Yes - 10 (20%) of cases	Change in posterior malleolus fixation in 9 cases and Chaput fracture in 1 case	IV
Black et al. (2013) [26]	100	Malleolar ankle fractures (AO type 44) treated operatively	Yes - 24% of cases, the management plan changed after CT evaluation. Predictors of change: tri-malleolar vs unimalleolar (29% vs 10%), preoperative dislocation (31% vs 20%), supra-syndesmotic fractures (40%)	Changes included fixation of the medial malleolus (21%), the posterior malleolus (15%), and occult anterolateral plafond fractures (9%)	III
Comadoll et al. (2024) [27]	288 (94 with CT)	Tri-malleolar fractures	No - No significant difference observed in rate of posterior malleolus fixation between groups (43.8% without CT vs 39.4% with CT; p=0.52)	Average surgical time was higher in patients without preoperative CT (114 minutes) than with CT (145 minutes; p<0.05). Complications (10.3% no CT vs 7.4% with CT, p=0.55) and reoperations (6.7% without CT vs 7.4% with CT, p=0.16) were not significantly different between groups	III

Across many studies, CT imaging was frequently associated with changes in surgical management [19-26]. Most changes involved adjustments to fixation strategy, fragment-specific approaches, and patient positioning, particularly in cases involving the posterior malleolus and complex tri-malleolar fractures.

Kumar et al., Meijer et al., and Kalantar et al. each noted similar rates of change in surgical approach following CT (23.2%, 23%, and 23.3%, respectively), reflecting its consistent role in preoperative planning [19,20,23]. Donohoe et al. reported the highest rate of management change following a CT (44%), mainly due to the detection of posterior fragments, which required modifications to the operative approach and position [21]. Likewise, Leung et al. described a 20% change in surgical management following CT, again predominantly for fixation of the posterior malleolus [25]. Collectively, these studies highlight the role of CT in optimising fragment exposure and fracture reduction.

For fracture-specific patterns, Massri-Pugin et al. showed that, in Tillaux-Chaput fractures (avulsion fracture of the anterolateral part of the tibial plafond), surgical decision changed in 12.5% of cases after CT classification alongside positioning (32.1%), fixation type (43.8%), and surgical approach (35.3%) [22]. Sheikh et al. reported CT-driven change in posterior malleolus (25.6%) and syndesmosis (16.6%) fixation in 32.7% of cases [24]. At the same time, Black et al. noted a 24% rate of modification in fixation strategy, with the highest rates in tri-malleolar (29%) and supra-syndesmotic fractures (40%) [26].

Conversely, Comadoll et al. found no significant difference in fixation rates between CT and non-CT groups for fixation of the posterior malleolus (43.8% without CT vs 39.4% with CT; p=0.52). Although operative time was longer with CT (145 vs 114 minutes, p<0.05), complication (10.3% without CT vs 7.4% with CT, p=0.55) and reoperation (6.7% without CT vs 7.4% with CT, p=0.16) rates were comparable [27].

Overall, these studies have shown that CT frequently revised surgical strategy; approximately one in four cases, particularly in complex fracture patterns such as posterior malleolus fractures, a region highlighted in most studies evaluating management change.

Discussion

Diagnostic Value of CT in Ankle Fractures

Across recent studies, CT consistently demonstrated superior diagnostic accuracy and anatomical characterisation of ankle fractures to X-ray.

A synthesis of the included studies indicates that conventional X-rays commonly miss findings, including posterior malleolar, syndesmotic, and intra-articular injuries. In contrast, CT provided enhanced visualisation of fracture morphology, displacement, and articular congruity [8,14,17]. By providing anatomical clarity, CT could facilitate accurate fracture classification. Bilateral or multi-planar CT was associated with better reduction assessment and may have implications for functional outcomes, although this evidence is observational and limited [10]. Overall, CT adds diagnostic value in cases with inconclusive X-rays, complex fracture patterns, or suspected articular involvement.

Impact of CT on Surgical Management and Planning

CT influenced surgical management in reported rates ranging from 12.5% to 44% across eight studies, primarily by clarifying fragment size, articular extension, and syndesmotic stability. This effect was most evident in tri-malleolar and posterior malleolus fractures, where CT characterisation dictated fixation strategy, surgical approach, and patient positioning. While several studies reported similar rates of change (around 20-25%), this consistency should be interpreted cautiously, given heterogeneity in definitions of “change in management” and overlapping cohorts [19,20,23,25]. However, despite CT’s diagnostic advantage, current evidence does not demonstrate consistent improvement in long-term functional outcomes or complication rates with CT-guided planning [27]. Thus, CT’s role appears mostly beneficial for anatomically complex injuries, where X-rays alone do not adequately guide fixation or approach.

Context Within Current Guidelines

This review aligns with current imaging recommendations by NICE (NG38) and the ACR Appropriateness Criteria (2020) [6,7]. Both bodies endorse selective, not routine, use of CT in acute ankle trauma. Whilst CT’s diagnostic superiority is acknowledged, there is insufficient evidence of improved outcomes, radiation balance, and cost-effectiveness. By consolidating post-NG38 evidence, this review identifies specific scenarios in which CT meaningfully altered management (tri-malleolar, posterior malleolar, syndesmotic, and X-ray-negative injuries), while reinforcing the limited value in simple or stable fracture configurations.

Proposed Selective CT Algorithm

**Figure 1:**
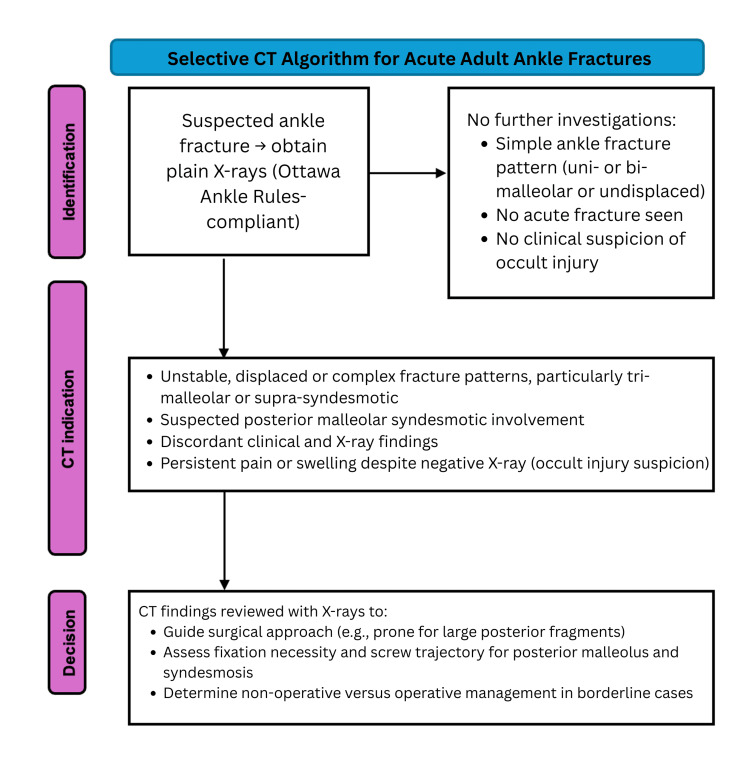
Proposed CT algorithm for acute adult ankle fractures CT: computed tomography

Based on the synthesis of findings and guideline gaps, a selective CT algorithm is proposed. The following steps outline this algorithm (Figure 1). This starts with initial imaging in the form of standard plain X-rays (Ottawa Ankle Rules-compliant) for suspected fractures. Any of the following found on X-ray or in history then warrant a further CT: unstable, displaced, or complex fracture patterns, particularly tri-malleolar or supra-syndesmotic; suspected posterior malleolar or syndesmotic involvement; discordant clinical and X-ray findings; persistent pain or swelling despite negative X-ray (occult injury suspicion). CT findings are then reviewed with X-rays to guide surgical approach (e.g., prone for large posterior fragments), fixation necessity, and screw trajectory for the posterior malleolus and syndesmosis, and to determine non-operative vs operative management in borderline cases.

This targeted approach balances diagnostic accuracy, radiation exposure, and cost, aligning with ACR and NICE caution against indiscriminate CT use [6,7].

Limitations

As a narrative review, the work is subject to selection and interpretation bias. Additional limitations include the use of a single database (PubMed), English-language restriction, and heterogeneity in outcome definitions - particularly “change in management.” Most included studies were retrospective, small, and single-centred (level III-IV evidence). Comparative cost-effectiveness and long-term functional outcomes remain unexplored. Larger prospective, multicentre trials are needed to determine whether CT-guided planning improves patient-centred outcomes and healthcare efficiency. The ALIGN trial (ISRCTN 25963775), which explores the use of CT in the assessment and management of ankle fractures, may help address this evidence gap [28].

## Conclusions

CT enhances anatomical understanding and surgical planning in complex ankle fractures, altering management in approximately 12.5-44% of cases. While its diagnostic and planning advantages are well established, evidence of improved, long-term patient outcomes remains limited and mostly observational. This review synthesises recent evidence to outline the impact of CT imaging on surgical decision-making and proposes a selective algorithm aligned with current guidance. The approach promotes targeted, cost-conscious imaging for cases where X-rays are insufficient until higher-level outcome data become available. Limitations of this review include reliance on a single database, heterogeneity in outcome definitions, and predominance of small, retrospective studies. Larger prospective trials are needed to determine whether CT-guided planning translates into measurable improvements in patient outcomes and healthcare efficiency.
